# Intensive exposure to narrative in story books as a possibly effective treatment of social perspective-taking in schoolchildren with autism

**DOI:** 10.3389/fpsyg.2014.00002

**Published:** 2014-01-16

**Authors:** Kohei Tsunemi, Ayana Tamura, Shino Ogawa, Tomoko Isomura, Hiroyasu Ito, Misako Ida, Nobuo Masataka

**Affiliations:** ^1^Iwaki Junior CollegeIwaki, Japan; ^2^Showa Women’s UniversityTokyo, Japan; ^3^Primate Research Institute, Kyoto UniversityInuyama, Japan

**Keywords:** autism, narrative, social perspective-taking, mental state, treatment

## Abstract

One of the major characteristics of autism is impairment of communication and socialization. While such impairment *per se* has been well documented, research into effective interventions for children with this developmental disorder is still limited. Here we present preliminary evidence for the possibility of improvement of the capability of social perspective-taking in schoolchildren with autism by having intensive experience with narrative, in which they were exposed to narrative in story books read by their parents over a consecutive 5- to 6-day-period. When their capability was evaluated on the basis of a conventional role-taking task, the mean score tended to increase after the exposure as compared to before the exposure, whereas such a change was not recorded in children who did not experience such exposure. These effects were confirmed when the children were retested 4 months later. Although preliminary, the current study represents a step toward the development of more effective social perspective-taking interventions for children with autism.

## INTRODUCTION

Particular characteristics of childhood autism involve a profound impairment of communication and language, which is occasionally noticeable despite an adequate capacity for speech among the more intellectually able children with this disorder (Sigman and Capps, 1997; Howlin et al., 1999; American Psychiatric Association, 2013). Pioneering studies have revealed the fact that those children have characteristic difficulties with personal pronouns and with relative terms for time and space, i.e., with the use of deictic terms (see Baron-Cohen et al., 2000; Markram and Markram, 2010 for review). A speaker must be sensitive to a hearer’s information needs in order to effectively communicate using such deictic terms, but even intellectually able adults with autism have difficulty achieving that, mainly due to impairment of the perspective-taking capability that is involved in the use of deictic terminology (Masataka, 2003).

Perspective-taking is an area of human functioning that entails a complex repertoire of linguistic and relational behavior. Cognitive and developmental psychologists commonly agree that the capability to take the perspective of another person greatly contributes to an individual’s success in social situations, and involves a critical and complex set of skills (Barnes-Holmes et al., 2004). Perspective-taking involves inferring another person’s desire and beliefs, in order to interpret their behavior and predict what they will do next (Sigman and Capps, 1997; Howlin et al., 1999). Common human activities that are believed to involve perspective-taking include deception, empathy, self-consciousness, self-reflection, persuasion, and pretence, as well as being essential for effective communication (Howlin et al., 1999).

Conceptually, perspective-taking can be classified into three levels, i.e., the perceptual level (comprehension of how others perceive the world one also perceives; Piaget and Inhelder, 1956), the cognitive level (comprehension of others’ personal knowledge and belief; Frith, 2003) and the social level (comprehension of others’ emotion and thought; Mar, 2004). Impairment in perspective-taking capability in children with autism involves each of these three levels (Reed and Peterson, 1990; Hamilton et al., 2009). Nevertheless, studies of autism conducted thus far have overwhelmingly concentrated on impairment at the cognitive level (typically known as the theory of mind problem, ToM). ToM is a term for a set of complex cognitive processes, enabled by a system of cognitive mechanisms, which result in “the ability to infer the mental state of others (e.g., their knowledge, intentions, beliefs, and desires)” (Ozonoff and Miller, 1995, p.417). Consequently, a great number of investigations on cognitive skill or ToM treatments have been conducted with children, particularly preschool children, with autism since the pioneering work by Ozonoff and Miller (1995), who attempted to develop perspective-taking in nine adolescents with autism in the context of a social skills training program. In the study, five of them in the treatment condition received specific instruction in perspective-taking strategies, while the remaining four (control group) received regular social skills training only. A variety of techniques were employed to teach perspective-taking skills, including role-play and video feedback. At the end of the experiment, 80% of the intervention group improved their ToM composite score, whereas only 25% of the control group did, but no effect was seen on parent or teacher ratings of participant social skills.

Further studies have also shown that children with autism can be taught to pass a ToM test said to be indicative of perspective-taking (e.g., Swettenham, 1996; Hadwin et al., 1997). Recently, some children with this disorder have participated in studies of the more basic component skill of perspective-taking (at the perceptual level; Heagle and Rehfeldt, 2006; Gould et al., 2011). Concerning social perspective-taking, however, there have been virtually no reports of such investigations, though understanding the thoughts, feelings and motivations of others is crucial to successful relationships. Moreover, although social perspective-taking is particularly important for children in the classroom, most research on intervention for children with autism has focused on the evaluation of its effects on preschool children. The general consensus among researchers is that social perspective-taking can influence many elements of the academic experience and that students who get higher grades also tend to be more motivated and more accurate in their perspective-taking as revealed by recent studies (McKenzie et al., 2010, 2011) that reported that adolescents with autism were substantially less influenced than neurotypical adolescents in their conditional reasoning by the presentation of contextual cues in the form of alternative and additional arguments. On the basis of this evidence, those authors hypothesized that the participants with autism were less likely to integrate relevant and available contextual knowledge into the reasoning process, which is closely associated with the ability of perspective-taking.

Here, we report the results of our preliminary attempts to provide brief social perspective-taking treatment consisting of telling original narratives in story books. Entertainment by looking at and listening to story books occurs ubiquitously in young children and their caregivers (Aram and Aviram, 2009). Children are considered to experience a variety of emotions through such opportunities, to acquire literacy skills as well as communication ability, and to cultivate imagery capability (Foorman and Torgesen, 2001; McGee and Morrow, 2005). A narrative is a depiction of events, which are driven by the intentional behaviors of agents with unique goals, in imagined settings that can parallel the real world (Mar, 2004). Understanding a narrative requires one to understand the intentions, goals, emotions as well as other mental state of characters, which is known as mentalizing (Frith and Frith, 2003). For narrative comprehension, one must take the perspective of a character and mentally represent his/her emotional state. This is the essential process of successful social perspective-taking.

Therefore, here we hypothesized that abundant experience with such narrative comprehension would enhance the capability of social perspective-taking in children with autism who show impairment in this cognitive aspect. We conducted the present treatment on the basis of the assumption that if children with autism come to achieve successful comprehension of more or less imagery sentences in story books through our treatment, their ability of processing the linguistic content will be enhanced to determine what is to be mentally imagined, and then the mental image can be evaluated and related to the sentence more properly by the children. Consequently, such experience would lead the children to enhance their capability of social perspective-taking. In fact, our findings provide suggestive evidence supporting the hypothesis that the ability to understand mental states of others could be improved by providing children with autism with opportunities to comprehend the activities of characters depicted in stories.

## MATERIALS AND METHODS

This investigation was conducted according to the principles expressed in the Declaration of Helsinki. All experimental protocols were consistent with the Guide for Experimentation with Humans and were approved by the Institutional Ethics Committee of the Primate Research Institute, Kyoto University (#2011-150). We obtained written informed consent from the parents of all participants involved in our study.

### PARTICIPANTS AND OVERALL DESIGN OF THE PRESENT STUDY

The participants consisted of 16 schoolchildren with autism and either of their mothers or fathers (14 mothers and two fathers). All of them spoke Japanese as their first language. The children had been diagnosed as such according to DSM-V (American Psychiatric Association, 2013) by psychiatrists from several facilities in Kyoto Prefecture, Japan. The general cognitive ability of the participants was assessed using Wechsler Intelligence Scale for Children (WISC III). The children were randomly classified into two groups, i.e., the group referred to as the Experimental Group below or that referred to as the Control Group below. Every child, whether classified into the Experimental Group or into the Control Group, was tested about his/her ability of perspective-taking twice in the 5- to 6-day-long experiment, i.e., at the beginning of the first day of the experiment and at the end of the final day of the experiment. During the experimental period, each child of the Experimental Group received intensive exposure to narrative as a treatment, but no such exposure was experienced by the children of the Control Group. Scores on an intelligence quotient (IQ) test, as well as the mean chronological age, did not differ significantly between the groups, as shown in **Table 1**.

**Table 1:** Mean chronological ages (years: months) and mean WISC IQ scores of the participants.

	Group		Significance of difference (*P*)
	Experimental (*N* = 9)	Control (*N* = 7)
Age	9:7 (1:0)	9:6 (1:5)	0.28
WISC III
Verbal IQ	106.2 (16.2)	108.6 (20.7)	0.26
Performance IQ	100.3 (17.7)	105.1 (12.6)	0.14
Full-scale IQ	103.9 (17.7)	107.6 (17.2)	0.92

*Standard deviations presented in parentheses. Probabilities were evaluated by *t*-tests (*df* = 14).
*

### PROCEDURE OF EXPOSURE TO NARRATIVE

For the treatment, we created a set of eight original story books in Japanese. The narrative of each of the eight was totally different from the others. However, each consisted of three episodes. The English version of the first episode of a representative narrative was as follows:

There was a boy named Taichi, who was attracted by creatures. One day, he found a frog on his way to school, caught it, and took it with him to school. After he entered his classroom, one of his classmates, Sayoko, came to him, and asked what Taichi had. Being flattered by her question, he showed her the frog. But she was scared of it, screamed, and started crying. Having heard the crying, their teacher appeared, and warned Taichi not to scare any friends and make them cry. Taichi was very depressed by what had happened. Returning back home, he visited a rice field to release his frog, and there he met another friend, Tamayo. She asked him what he had in his hands. Hearing that question, he really felt embarrassed.

Each time the parent finished telling the first episode of a narrative, he/she was instructed to ask his/her child some questions concerning the mental states of the characters depicted in the episode, and some example questions were actually suggested.

With regard to the representative story mentioned above, they were:

(1) Why was Taichi embarrassed by Tamayo’s question?
(2) If Taichi shows the frog to Tamayo, how will she respond to it?
(3) If Taichi had talked with Sayoko about the frog instead of suddenly showing it to her, what do you expect would have happened with the two of them? I am wondering if you can guess how both of them would have felt.
  These questions were open-ended and had many possible proper answers. Parents were told to praise their children no matter how they responded to the questions. Following these questions, the second episode, which was as follows, was related:
  One day, when Sayoko entered the classroom, her classmate Taichi had something in his hands. She got interested in what he had, and asked him to open his hands. When she saw what he showed, she was very scared of the frog, and cried because she was scared of it. However, she felt sorry for Taichi when he was scolded by the teacher and looked depressed.

While the first episode was told from the viewpoint of *Taichi*, the second one was apparently told from the viewpoint of *Sayoko*.

Having finished reading the second episode, the parents continued by reading the final one after a brief break. This episode was as follows:

*Taichi told Tamayo that there was a frog in his hand, and asked whether she loved or hated frogs. Having confirmed that* *she loved frogs, he showed the frog to her. They played with the frog for a while and released it in a rice field. There, Sayoko came out, and apologized to Taichi about the frog. Taichi forgave Sayoko and they went home together.*

Obviously this episode was prepared as an “answer” to the final question of the three the parents asked after telling the first episode. Parents of the participants were asked to read some of the narrative to the participant every day for a roughly 30-min period any time in the day during a 5- to 6-day period in August. Note that the treatment actually lasted only these 5–6 days.

They were given standardized instructions which were as follows: The purpose is to have your child imagine various perspectives and emotions of characters which are depicted in the narrative. Depending on the ability and motivation of your child, you may read to a child, or the child herself/himself may read aloud/silently. The narrative also includes several questions you should ask to your child. You are allowed to converse with your child about impressions about the narrative. If your child unavoidably loses her/his concentration, you need not force him/her to keep reading, but should try again at some other time.

During the experimental period, each parent was asked to keep detailed records about their practice of the exposure to narrative so that we could confirm that the treatment was really undertaken every day.

### ASSESSMENT OF THE ABILITY OF PERSPECTIVE-TAKING

Just before the onset of the experiment and upon the completion of the entire experiment, each participant of the Experimental Group was assessed with respect to their competence of perspective-taking (perceptually, cognitively, as well as socially). The same assessment was also made twice for the participants of the Control Group, with the same interval between assessments as that for the participants of the Experimental Group. However, no exposure was conducted during the interval.

The assessment consisted of three tasks to investigate capability of perspective-taking: perceptual, cognitive and social perspective-taking tasks. The capability of perspective-taking at the perceptual level was evaluated on the basis of the three-mountain-task (Piaget and Inhelder, 1956). We prepared four photographs of three blocks, different in shape and colors, which changed in appearance depending on the viewer’s perspective. The Experimenter presented one of the photographs on a personal computer, and asked children to guess how others would see it from their viewpoint, and to choose the proper one from the other three photographs by pointing to it. Children were asked twice about the appearance of the right and left side, and when they correctly answered both times, they considered to have passed the task.

The capability of perspective-taking at the cognitive level was evaluated on the basis of the false belief task (secondary level, Perner and Wimmer, 1985). Perner and Wimmer’s (1985) second-order false belief test requires recursive understanding of one story character’s belief about another’s belief. Four pictures were presented on a personal computer screen. The Experimenter read aloud the task and asked the children to answer three questions. The first was a question regarding the secondary level of false belief: Where the boy thought the girl thought that the toy was? The two probe questions followed: where the toy actually was, and where the toy was put by the girl at first. Children who correctly answered all three questions were regarded to have passed the task.

For each task, the performance was given a score of points ranging from 0 to 1 for each individual. Children received a “1” if they passed the task and a “0” if they failed the task. On the basis of this score, the average value was computed both for the Experimental Group and for the Control Group before and after the treatment.

Regarding social perspective-taking, the capability of each child was evaluated using a Japanese version of a role-taking task originally developed by Selman (1980). Each of the items of the task consists of a set of drawings and narrative. The English version of a representative narrative is as follows (each number corresponds to one of several different drawings that are to be presented simultaneously):

(1) Once upon a time, there was a girl named Junko, who was good at climbing trees. One day, though she tried to climb down a tree, Junko failed to do so and fell down to the ground.
(2) Her father happened to watch it and scolded her so that Junko was forced to promise that she would never climb any tree.
(3) A few days later, it was found that a cat kept by Junko’s neighbor, Taro, had climbed up the tree, and remained there helplessly. But Taro was poor at climbing trees, and asked Junko to help his cat.
(4) Unfortunately, no one was around there, and the only person who could help the cat was Junko, who was good at climbing trees. She was embarrassed by remembering her promise made to her father a few days earlier.

After the narrative, the participant was asked to answer the following questions verbally:

(1) Why was Junko embarrassed?
(2) If Junko decides to climb up the tree to help the cat, what do you expect her father to do?
(3) What is the reason for his response?
(4) What do you expect regarding the future of the cat if Junko and her father discuss the cat?

All the answers given by all the participants were tape-recorded and transcribed later. These verbal responses were rated by two experimenters, independently, into one of three categories, reflecting three assumed levels of role-taking ability: 1 (differentiated and subjective perspective-taking: concepts of persons are differentiated, but concepts of relations stay subjective), 2 (self-reflective/second-person and reciprocal perspective: one can take a self-reflective or second-person perspective, and consider concepts of reciprocal relationship of self and another), and 3 (third-person and mutual perspective-taking: one can take a third-person perspective, and consider simultaneous and mutual coordination of self and others). When the rates’ scores were different from one another between the raters, they discussed it and decided on the final score. Thereafter, the scores for each of the four questions were averaged for the first measurement and for the second measurement, and the mean value was computed for each measurement in the Experimental and in the Control Groups.

## RESULTS

On the basis of the parental records, it was found that each participant child in the Experimental Group experienced the exposure to the narrative in a total of 10.4 times on average over the treatment period. As noted above, the purpose of the present study was to evaluate the effects of these experiences upon the capability of social perspective-taking in the schoolchildren with autism, using a conventional role-taking task. Actual transcriptions of all of the participants’ answers to question 4 of the role-taking task (Selman, 1980) in the first and the second measurements that is documented in METHODS are shown in **Table 2**. As found there, noticeable changes in the answer between the first and the second measurements were seen in four of the nine children in the Experimental Group, whereas similar changes occurred in one of the seven children in the Control Group. Indeed, concerning these four children in the Experimental Group and one child in the Control Group, the overall score did show elevation from 1 (differentiated and subjective perspective-taking: concepts of persons are differentiated, however, concepts of relations stay subjective) to 2 (self-reflective/second-person and reciprocal perspective: one can take self-reflective or second-person perspective, and consider concepts of reciprocal relationship of self and another), or from 2 to 3 (third-person and mutual perspective-taking: one can take third-person perspective, and consider simultaneous and mutual coordination of self and others), whereas no such elevation of the score was recorded for any other participant. One child of the Control Group even showed a decrease of the score from 3 to 2.

**Table 2:** Comparisons of transcriptions of the verbal responses between the first and the second measurements that were recorded to the question “*How do you expect the story of the cat to turn out if Junko and her father discuss the cat?* ” which was asked in the inventory of social perspective-taking.

Group	Participant code	1st	2nd
Experimental	A*	Junko will climb up the tree.	The cat will helped by Junko.
	B*	The cat will be helped by Junko.	I think she should not climb up because it is dangerous.
	C	The cat will be helped by a fire truck equipped with a ladder.	Her father will permit Junko to help the cat if she takes adequate care.
	D*	Junko should have climbed with the help of a ladder.	Her father will help the cat with the use of a ladder.
	E	Having no other option, Junko will climb up the tree.	Her father will permit Junko to climb up the tree and take the cat down.
	F	Her father will bring a ladder, climb on it, and help the cat.	Her father will ask someone to bring a ladder, climb on it and help the cat.
	G	They will discuss the way to help the cat.	The cat should have been saved. Maybe her father will be able to climb.
	H	Her father helped the cat!	Her father will attempt to help the cat, maybe, by climbing on something like a ladder.
	I*	I think the cat will possibly be helped because Junko will climb up the tree.	I think the cat will be helped by Junko’s climbing up the tree.
Control	J	Junko will climb up the tree, and her father will keep watching her actions.	The cat will be helped by Junko.
	K	Her father will climb up the tree.	Junko will warn the cat about its action, and ask her father for permission to climb up to it.
	L	I believe her father will permit Junko to climb. Otherwise, he will climb up by himself.	I think Junko would help the cat.
	M+	I think her father will call for a fire truck.	Her father will change his mind and permit calling for a fire truck.
	N*	Junko will be permitted to climb up the tree.	As she promised her father, helping the cat will be OK.
	O	Her father will allow her to help the cat.	The cat will be OK because Junko will help it.
	P	Her father will bring a ladder and help the cat.	Her father will bring a ladder and help the cat.

*Asterisk denotes participants whose scores increased at the second testing, + denotes participant whose scores decreased at the second testing.
*

In all, among the nine children of the Experimental Group, the number of the children who showed the elevation of the score was 4 whereas that of the Control Group was 1. The number of children who did not show any change was 5 in both of the groups. None of the children showed a decrease of the score in the Experimental Groups whereas one child of the Control Group showed such decrease. When within-group changes were evaluated on the basis of these results, using Sign tests, the increase of the overall rated scores of the role-taking task tended to be significant (*p* = 0.063) for the participants of the Experimental Group, but not for those of the Control Group (*p* = 0.75).

With regard to the three-mountain task (Piaget and Inhelder, 1956), on the other hand, mean scores (SDs) at the first measurement were 1.00 (0.00) for the Experimental Group and 0.86 (0.38) for the Control Group. The difference of the scores between the groups was not statistically significant (*p =* 0.35). At the second measurement, mean scores (SDs) were 0.89 (0.34) for the Experimental Group and 1.00 (0.00) for the Control Group. The difference between them was not significant (*p =* 0.36), either. With regard to the false belief task (Perner and Wimmer, 1985), mean scores (SDs) at the first measurement were 0.78 (0.45) for the Experimental Group and 0.58 (0.54) for the Control Group. The difference between the scores was not statistically significant (*p =* 0.68). At the second measurement, mean scores (SDs) were 0.67 (0.50) for the Experimental Group and 0.72 (0.49) for the Control Group. Again, the difference was not significant (*p =* 0.36).

We also evaluated the ability of social perspective-taking of all the participants except one child in the Experimental Group once again at 40 days after the testing that was performed immediately after the intense treatment, using the same inventory. As shown in **Table 3**, however, their scores did not exhibit substantial changes, indicating the long-term stability of the changes evoked by the treatment.

**Table 3:** Comparisons of transcriptions of the verbal responses between the first and the third measurements that were recorded to the question “*How do you expect the story of the cat to turn out if Junko and her father discuss the cat?* ” which was asked in the inventory of social perspective-taking.

Group	Participant code	1st	3rd
Experimental	A*	Junko will climb up the tree.	Junko had permission from her father to climb the tree.
	B	The cat will be helped by Junko.	–
	C*	The cat will be helped by a fire truck equipped with a ladder.	I believe her father will permit Junko to climb. Otherwise, he will call for a fire truck.
	D*	Junko should have climbed with the help of a ladder.	Her father will help the cat with a ladder.
	E	Having no other option, Junko will climb up the tree.	Having no other option, her father will allow her to climb the tree.
	F	Her father will bring a ladder, climb on it, and help the cat.	Her father will bring a ladder and help the cat.
	G*	They will discuss the way to help the cat.	Her father will help the cat.
	H	Her father helped the cat!	Her father will help the cat somehow.
	I*	I think the cat will possibly be helped because Junko will climb up the tree.	Junko will climb the tree and help the cat.
Control	J	Junko will climb up the tree, and her father will keep watching her actions.	Junko will be permitted to climb up the tree just for once.
	K+	Her father will climb up the tree.	Junko will be permitted to climb up the tree with grown-up.
	L*	I believe her father will permit Junko to climb. Otherwise, he will climb up by himself.	Junko may be permitted to climb up the tree. If she couldn’t, then call a friend who is good at climbing.
	M	I think her father will call for a fire truck.	Junko call for a fire truck or a neighbor, or wait for the cat climb down, or encourage it.
	N	Junko will be permitted to climb up the tree.	–
	O	Her father will allow her to help the cat.	The cat will be helped. Junko will talk her father that she wants to help the cat which cannot get down. Then he will allow her to climb the tree and she will climb to help it.
	P	Her father will bring a ladder and help the cat.	I think that her father will bring a ladder and make it through.

*Asterisk denotes participants whose scores increased at the third testing, + denotes participant whose scores decreased at the third testing.*

## DISCUSSION

The finding of the present experiment that the average rating score was increased between the first and second measurements only by the role-taking task (Selman, 1980), which measures the performance of social perspective-taking, exclusively in those participants who experienced exposure to narrative indicates the effectiveness of this exposure as a treatment to promote such ability. The stability of the change of the performance was also confirmed by the results of retesting which was performed 4 months later.

Obviously, the cognitive process underlying narrative comprehension is a complicated one. First, it requires one to understand intensions, goals, emotions, and some other variable mental states of the characters (Gernbacher et al., 1992; de Vega et al., 1996; Komeda and Kusumi, 2006). If narrative comprehension involves social cognitive processes, then we would expect individuals who frequently engage with narratives to benefit socially in some way from these repeated experiences. Namely, with the exposure to narrative, one needs to take the perspective of a character and to mentally represent his or her emotional state to understand the behavior of the character, while social perspective-taking involves the capability of adopting and understanding the perspective of others, on the basis of which one empathizes with their experience and reactions (Davis, 1983; Blair, 2005). A recent study (Mar et al., 2006) indeed demonstrated that lifetime exposure to narrative fiction, controlling for exposure to expository non-fiction, is positively associated with social abilities, though the correlational nature of that study excludes the possibility of any causal inference. Subsequently, additional evidence was presented supporting the notion that there is a positive relation between exposure to story books and social development in preschool children (Aram and Aviram, 2009; Mar et al., 2010).

The present attempt to provide intensive exposure of schoolchildren with autism to narrative was conceived in order to pursue this issue further, and to apply such previous findings for the actual treatment for those children. In fact, the present findings suggest the possibility that in order to cultivate competence in social perspective-taking, intensive experience with story-telling over a period of some length would be effective. As an actual protocol of the intervention, moreover, importance of some open-ended questions the parents were instructed to ask each time they finished telling an episode should also not be dismissed. Because their performance of story-telling *per se* is likely to merely provide their children with opportunities to attend to a series of behaviors of characters depicted there, those questions could more actively solicit the children to understand the thoughts feelings and motivation of the characters.

Traditionally, the visuospatial system has been viewed as an intact area in people with autism, as represented by the phrase, “they are thinking in pictures much of the time” (Grandin, 1995). Typically, they perform better on a picture-word completion task than on a word–word completion task, suggesting an advantage of visual images over linguistic representation in access to semantics in autism. Recently, such a phenomenon has been explained in terms of an underlying neurological under connectivity among cortical areas in autism (Just et al., 2004), which could negatively impact or slow integration or communication among cortical regions involved in language and visual imagery processing. This explanation attributes many of the wide-spread abnormalities in psychological functioning in autism to an impairment in the coordination and communication between key brain processing centers. One of the main predictions made based on this explanation is that any facet of psychological and neurological function that is dependent on the coordination or integration of brain regions is susceptible to disruption in autism. That is particularly the case when the computational demand of the coordination is large, and social perspective-taking is no doubt included among such cases. The acknowledgment of the important role of visual thinking has led to the common use of treatment methods that are based on picture exchange communication (Bondy and Frost, 1998) or visual organizers such as the TEACCH method (Schopler et al., 1998). On the other hand, verbal communication has continued to be primarily limited to the expression of instrumental functions, or simple labeling. Consequently, the capability of social perspective-taking in people with autism has remained underdeveloped. In this regard, we hypothesize that what is necessary for effective treatment of this capability is to provide such people with opportunities to rely on visualization to support their comprehension of the thoughts, feelings and motivations of others that are represented by language. The present findings suggest that intensive exposure to narrative should be an effective method to accomplish that, and lead to the above hypothesis, which is worth testing with a broader sample size than that investigated here. If it is really confirmed, exposure to narrative would be expected to be a particularly convenient method of treatment for children with autism, possible by enhancing cortical connectivity, which is characteristically decreased in children with autism.

## Conflict of Interest Statement

The authors declare that the research was conducted in the absence of any commercial or financial relationships that could be construed as a potential conflict of interest.

## References

[B1] American Psychiatric Association. (2013). *Diagnosis and Statistical Manual of Mental Disorders* 5th Revised Edn. Washington D.C.: American Psychiatric Association

[B2] Aram D. Aviram S. (2009). Mothers’ storybook reading and kindergartners’ socioemotional and literacy development. *Read. Psychol.* 30 175–194 10.1080/02702710802275348

[B3] Barnes-Holmes Y. McHugh L. Barnes-Holmes D. (2004). Perspective-taking and theory of mind: a relational frame account. *Behav. Anal. Today* 5 1–25

[B4] Baron-Cohen S. Tager-Flusberg H. Cohen D. (2000). *Understanding Other Mind*. Oxford: Oxford University Press

[B5] Blair R. J. (2005). Responding to the emotions of others: dissociating forms of empathy through the study of typical and psychiatric populations. *Conscious. Cogn.* 14 698–718 10.1016/j.concog.2005.06.004 16157488

[B6] Bondy A. Frost L. (1998). The picture exchange communicating system. *Semin. Speech Lang.* 19 373–389 10.1055/s-2008-1064055 9857393

[B7] Davis M. H. (1983). Measuring individual differences in empathy: evidence for a multidimensional approach. *J. Person. Soc. Psychol.* 44 113–126 10.1037/0022-3514.44.1.113

[B8] de Vega M. Leon I. Diaz J. M. (1996). The representation of changing emotions in reading comprehension. *Cogn. Emot.* 10 303–321 10.1080/026999396380268

[B9] Foorman B. R. Torgesen J. (2001). Critical elements of classroom and small-group instruction promote reading success in all children. *Learn. Disab. Res. Pract.* 16 203–212 10.1111/0938-8982.00020

[B10] Frith U. (2003). *Autism: Explaining the Enigma*. Oxford: Blackwell

[B11] Frith U. Frith C. D. (2003). Development and neurophysiology of mentalizing. *Philos. Trans. R. Soc. Lond. B Biol. Sci.* 358 459–473 10.1098/rstb.2002.1218 12689373 PMC1693139

[B12] Gernbacher M. A. Goldsmith H. H Robertson R. R. W. (1992). Do readers mentally represent character’s emotional states? *Cogn. Emot.* 6 89–111 10.1080/02699939208411061 PMC4191745 25309018

[B13] Gould E. Tarbox J. O’Hara D. Noone S. Bergstrom R. (2011). Teaching children with autism a basic component skill of perspective-taking. *Behav. Intervent.* 26 50–66 10.1002/bin.320

[B14] Grandin T. (1995). *Thinking in Pictures: And Other Reports from My Life*. New York: Doubleday

[B15] Hadwin J. Baron-Cohen S. Howlin P. Hill K. (1997). Does teaching theory of mind have an effect on the ability of develop conversation in children with autism? *J. Autism Dev. Disord*. 5 519–538 10.1023/A:1025826009731 9403370

[B16] Hamilton A. F. Brindley R. Frith U. (2009). Visual perspective taking impairment in children with autistic spectrum disorder. *Cognition* 113 37–44 10.1016/j.cognition.2009.07.007 19682673

[B17] Heagle A. I. Rehfeldt R. A. (2006). Teaching perspective-taking skills to typically developing children through derived relational responding. *J. Early Intens. Behav. Interv.* 3 1–34

[B18] Howlin P. Baron-Cohen S. Hadwin J. (1999). *Teaching Children with Autism to Mind-Read*. New York: Wiley

[B19] Just M. A. Cherkassky V. L. Keller T. A. Minshew N. J. (2004). Cortical activation, synchronization during sentence comprehension in high-functioning autism: evidence of under connectivity. *Brain* 127 1811–1821 10.1093/brain/awh199 15215213

[B20] Komeda H. Kusumi T. (2006). The effect of a protagonist’s emotional shift on situation model construction. *Mem. Cognit.* 34 1548–1556 10.3758/BF03195918 17263078

[B21] Mar R. A. (2004). The neuropsychology of narrative: story comprehension, story production and their interrelation. *Neuropsychology* 42 1314–1434 10.1016/j.neuropsychologia.2003.12.016 15193948

[B22] Mar R. A. Oatley K. hirsh J. dela Paz J. Peterson J. B. (2006). Bookworms versus nerds: exposure to fiction versus nonfiction, divergent associations with social ability, and the stimulation of fictional social worlds. *J. Res. Personal.* 40 694–712 10.1016/j.jrp.2005.08.002

[B23] Mar R. A. Tackett J. L. Moore C. (2010). Exposure to media and theory-of-mind development in preschoolers. *Cogn. Dev.* 25 69–78 10.1016/j.cogdev.2009.11.002

[B24] Markram K. Markram H. (2010). The intense world theory – a unifying theory of the neurobiology of autism. *Front. Hum. Neurosci.* 4:224 10.3389/fnhum.2010.00224 PMC3010743 21191475

[B25] Masataka N. (2003). *The Onset of Language*. Cambridge: Cambridge University Press 10.1017/CBO9780511489754

[B26] McGee L. M. Morrow L. M. (2005). *Teaching Literacy in Kindergarten*. New York: Guilford Press

[B27] McKenzie R. Evans J. S. B. T. Handley S. J. (2010). Conditional reasoning in autism: activation and integration of knowledge and belief. *Dev. Psychol.* 46 391–403 10.1037/a0017412 20210498

[B28] McKenzie R. Evans J. S. B. T. Handley S. J. (2011). Autism and performance on the suppression task: reasoning, context and complexity. *Think. Reason.* 17 182–196 10.1080/13546783.2010.549302

[B29] Ozonoff S. Miller J. N. (1995). Teaching theory of mind: a new approach to social skills training for individuals with autism. *J. Autism Dev. Disord.* 25 415 10.1007/BF02179376 7592252

[B30] Perner J. Wimmer H. (1985). “John thinks that Mary thinks that… ’’: attribution of second order beliefs by 5- to 10-year-old children. *J. Child Psychol.* 39 437–471 10.1016/0022-0965(85)90051-7

[B31] Piaget J. Inhelder B. (1956). *The Child’s Conception of Space*. London: Routledge

[B32] Reed T. Peterson C. (1990). A comparative study of autistic subjects’ performance at two levels of visual and cognitive perspective taking. *J. Autism Dev. Disord.* 20 555–567 10.1007/BF02216060 2279973

[B33] Schopler E. Reichler I. Renner B. R. (1998). *The Childhood Autism Rating Scale*. Los Angeles: CARS Wester Psychological Service

[B34] Selman R. L. (1980). *The Growth of Interpersonal Understanding: Developmental and Clinical Analyses*. New York: Academic Press

[B35] Sigman M. Capps L. (1997). *Children with Autism: A Developmental Perspective*. Cambridge: Harvard University Press

[B36] Swettenham J. (1996). Can children with autism be taught to understand false belief using computers? *J. Child Psychol. Psychiatry* 27 647–656 10.1111/j.1469-7610.1996.tb01387.x 8682895

